# Comparative Animal Study of Zirconia-Coated Titanium Implants: Effect on Bone Formation and Collagen Fiber Orientation

**DOI:** 10.3390/jfb16120449

**Published:** 2025-11-29

**Authors:** Kohei Osawa, Masatsugu Hirota, Toshitsugu Sakurai, Yohei Iinuma, Chikahiro Ohkubo, Hiroki Nagai, Takatsugu Yamamoto, Kenji Mitsudo

**Affiliations:** 1 Department of Oral and Maxillofacial Surgery/Orthodontics, Yokohama City University Medical Center, 4-57 Urafune-cho, Minami-ku, Yokohama 232-0024, Kanagawa, Japan; 2 Center of Oral and Maxillofacial Implantology, Tsurumi University Dental Hospital, 2-1-3, Tsurumi, Tsurumi-ku, Yokohama 230-8501, Kanagawa, Japan; okubo-c@tsurumi-u.ac.jp; 3 Department of Education for Dental Medicine, Tsurumi University School of Dental Medicine, 2-1-3, Tsurumi, Tsurumi-ku, Yokohama 230-8501, Kanagawa, Japan; hirota-masatsugu@tsurumi-u.ac.jp (M.H.); sakurai-toshitsugu@tsurumi-u.ac.jp (T.S.); yamamoto-tk@tsurumi-u.ac.jp (T.Y.); 4 Department of Oral Rehabilitation and Prosthodontics, Tsurumi University School of Dental Medicine, 2-1-3, Tsurumi, Tsurumi-ku, Yokohama 230-8501, Kanagawa, Japan; iinuma-y@tsurumi-u.ac.jp; 5 Department of Applied Physics, School of Advanced Engineering, Kogakuin University, 2665-1, Nakano-machi, Hachioji 192-0983, Tokyo, Japan; nagai@cc.kogakuin.ac.jp; 6 Department of Operative Dentistry, Tsurumi University School of Dental Medicine, 2-1-3, Tsurumi, Tsurumi-ku, Yokohama 230-8501, Kanagawa, Japan; 7 Department of Oral and Maxillofacial Surgery, Yokohama City University Graduate School of Medicine, 3-9, Fukuura, Kanazawa-ku, Yokohama 236-0004, Kanagawa, Japan; mitsudo@yokohama-cu.ac.jp

**Keywords:** zirconia-coated implant, molecular precursor method, bone, gingiva, collagen fiber

## Abstract

Tissue responses to zirconia-coated implants treated with molecular precursor method were evaluated. The zirconia film was characterized using scanning electron microscopy (SEM), atomic force microscopy (AFM), energy dispersive X-ray spectroscopy (EDX), X-ray photoelectron spectroscopy (XPS), and X-ray diffraction (XRD). Cylindrical titanium (ZrO_2_/Ti) specimens were sandblasted, acid-etched, and coated with zirconia using the molecular precursor method. Control specimens were sandblasted and acid-etched only (SLA/Ti). After maxillary first molar extraction, four ZrO_2_/Ti and four SLA/Ti implants were placed in the alveolar bone of the rats, and tissue responses were observed after 3 weeks. Surface analysis using SEM and AFM showed zirconia was present on ZrO_2_/Ti surface, with coating not affecting surface morphology compared to SLA/Ti. EDX, XPS, and XRD measurements confirmed the ZrO_2_ coating on the roughened Ti. The amount of new bone was greater in ZrO_2_/Ti (77.0 ± 7.2%) than in SLA/Ti (59.7 ± 5.8%) (*p* = 0.807). Collagen fibers oriented perpendicular to implant surface were observed more frequently in ZrO_2_/Ti (67.3 ± 9.5%) than in SLA/Ti (18.8 ± 10.01%) (*p* < 0.001). The area of perpendicular collagen fibers was significantly larger in ZrO_2_/Ti (53.1 ± 13.4%) than in SLA/Ti (16.8 ± 2.6%) *(p* = 0.002). Zirconia-coated implants maintained surface morphology and improved bone formation and fiber orientation in the gingiva compared to conventional titanium implants in short-term animal experiments.

## 1. Introduction

Titanium (Ti) is a material which is widely used for dental implants; however, in recent years, zirconia implants are attracting attention. In dental applications, partially stabilized zirconia (Y-TZP), made by adding a small amount of stabilizer such as yttrium to zirconia, is applied due to its superior flexural strength and toughness compared to other ceramics [1]. Key advantages of Y-TZP implants include favorable aesthetic properties, metal-free compositions (allowing their application in patients with metal allergies), and relatively low incidences of gingival inflammation after implantation [2]. Furthermore, the polished Y-TZP surface is smoother than that of Ti and is hydrophobic; as a result, plaque adheres to it less, resulting in lower peri-implant inflammation in the transgingival area [3]. However, few long-term clinical reports exist on Y-TZP as a dental implant. In addition, although Y-TZP has high toughness, it is brittle, leading to concerns about fracture and stress concentration in bone tissue compared to metallic materials. Another problem is that the extremely hard Y-TZP surface is difficult to machine or sandblast, such as by sandblasting, which is essential for achieving bone contact.

However, the clinical effectiveness of zirconia implants as an alternative to titanium implants remains controversial. Survival and success rates of zirconia implants were comparable to those of titanium implants at 12 months of follow-up [4]. In contrast, another review reported that the clinical outcomes of zirconia implants were similar or inferior to those of titanium implants [5]. Duan et al. compared the clinical performance of zirconium and titanium implants [6]. They reported that zirconium implants may have a lower survival rate and significantly lower success rate than titanium implants. Regarding the peri-implant, no difference was observed between titanium and zirconium implants in terms of marginal bone loss, probing pocket depth, bleeding on probing, plaque index, or pink esthetic score.

To avoid peri-implantitis, which destroys the bond between the implant and surrounding bone [7], it is important to control inflammation around the implant. Inflammation around implants progresses more quickly than inflammation around natural teeth [8]. One of the reasons for this is thought to be the difference in the course of collagen fiber bundles in the gingiva [9]. The collagen bundles in natural teeth are arranged vertically, whereas the collagen bundles in implants are arranged parallel [10]. Araujo et al. reported in their study that the lack of root cementum on the implant surface, and the difference in the orientation of the collagen fibers in soft tissue, may be associated with the variation observed in resistance to probing [11]. Therefore, we considered that the orientation of collagen fiber bundles perpendicular to the implant is an important factor in improving soft tissue adhesion. Previous studies have investigated the effects of Y-TZP implants on soft tissues and reported an increase in collagen fibers oriented perpendicular to the implant [12]. In this study, Y-TZP implant surfaces treated with a combination of large-grit sandblasting and hydrofluoric acid etching followed by ultraviolet irradiation were found to be effective in enhancing the perpendicular orientation of collagen fibers towards the implant body. However, the sandblasted surface of Y-TZP is completely different from that of titanium [13]. The arguments between Ti and Y-TZP implants on hard- and soft-tissue responses are based on different surface morphologies, including surface roughness. Therefore, the tissue responses of implants with the same or similar surface morphologies should be discussed. This made the characteristics of the zirconia surface more apparent.

Given the limitations of processing Y-TZP, in a past study, we opted to coat Ti with zirconia (ZrO_2_) [13]. Using Ti as the base material, this strategy aimed to obtain the advantages of the surface chemistry of ZrO_2_ while maintaining favorable mechanical properties of Ti. Kaluđerović et al. fabricated a ZrO_2_-coated titanium implant using anodic plasma-electrochemical oxidation technique [14]. However, the coated ZrO_2_ surface has a heterogeneous porous structure that is different from that of the starting titanium surface. We coated Ti disks with ZrO_2_ using a molecular precursor method and evaluated their effects on hard tissues in an extraoral environment [13]. The surface morphology and roughness of the base Ti material were maintained on the ZrO_2_ surface because of the thin film coating. The results revealed that the ZrO_2_-coated sample was effective with regard to promoting bone formation in the early stages of the bone-healing process, equal to or greater than that of Ti. In addition, because this was a thin-film coating, the surface morphology and roughness of the base material were maintained.

Building on these studies, for evaluating tissue response of ZrO_2_ surface in vivo oral environment, we use Ti as the implant body and create a ZrO_2_-coated implant in which only the surface is coated with ZrO_2_ in this study. This study aimed to evaluate the effects on hard and soft tissues of embedding cylindrical ZrO_2_-coated implants in the extraction sockets of maxillary molars in an actual oral environment in animal experiments. The evaluation of soft tissues focused mainly on collagen fiber bundles. The null hypothesis of this study was that ZrO_2_-coated Ti and Ti implants have the same effect on the response of the surrounding hard and soft tissues, especially on collagen orientation in the soft tissue region.

## 2. Materials and Methods

### 2.1. Specimen Preparation

**Figure 1:**
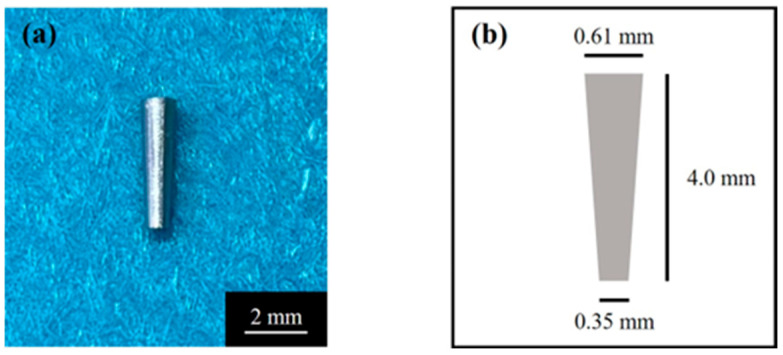
(**a**) Tapered cylindrical titanium implant and (**b**) its schematic.

The cylindrical implant body used is shown in Figure 1. A tapered cylindrical Ti sample (JIS2 type, ASTM grade 2, 99.9% mass, Nissin, Kyoto, Japan) with a certain conicity (upper diameter 0.61 mm, lower diameter 0.35 mm, length 4.0 mm) was manufactured using a computer-aided design/computer-aided manufacturing device (Aadva Mill LD-I, GC Corp., Tokyo, Japan) based on standard triangulation data. Ti disks (JIS2 type, ASTM grade 2, 99.9% mass, Furuuchi Chemical, Tokyo, Japan) (12.0 mm diameter, 1.0 mm thickness) were used for atomic elemental analysis and X-ray diffraction.

Before ZrO_2_ coating, the specimens were sandblasted and acid-etched (SLA). Sandblasting was performed perpendicular to the sample surface, using alumina particles with a size of 180 μm, at a distance of 20 mm from the sample, and with an air pressure of 0.6 N/mm^2^. Then, acid etching was performed on the blasted surface with a mixture of 36% hydrochloric acid (HCl) and a 96% sulfuric acid (H_2_SO_4_) solution for 3 min at 70 °C [15]. The SLA-treated specimens were then ultrasonically cleaned in ethanol and distilled water for 20 min (ultrasonic cleaner; VS-100III; AS ONE, Osaka, Japan).

**Figure 2:**
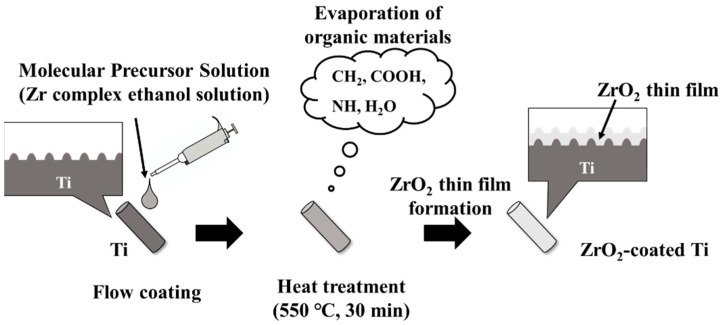
Schematic illustration for the coating process.

Ti implants were coated with ZrO_2_ (ZrO_2_/Ti) using the molecular precursor method [13,16,17]. The schematic illustration for the coating process is shown in Figure 2. 25 mL of a molecular precursor solution for the ZrO_2_ film (Zr complex ethanol solution; Zr^4+^ concentration = 0.32 mmol/g; TFTECH, Tokyo, Japan) was dropped onto the Ti surface. The cylinder samples were coated using flow coating, and the disk samples were coated using a spin coater (1H-D7, MIKASA, Tokyo, Japan) in a two-step mode: specifically, at 5 s 500 rpm, followed by 30 s 2000 rpm. Then, Ti implant specimens treated with precursor solution were then heat treated in a silica tube furnace (EPKPO12-K; ISUZU, Niigata, Japan) under atmospheric conditions. The heating temperature and time was 550 °C and 30 min, respectively. Thus, thin-film zirconia coatings can be obtained on Ti implants.

### 2.2. Surface Characterization of the Zirconia Coatings

Cylinder samples were characterized via scanning electron microscopy (SEM), atomic force microscopy (AFM), and energy dispersive X-ray spectroscopy (EDX) observation. Disk samples were for X-ray photoelectron spectroscopy (XPS) and X-ray diffraction (XRD) measurements. Contact angle measurements towards distilled water were performed on cylinder and disk samples.

The surface morphology of ZrO_2_/Ti was observed using SEM (SU1510; Hitachi High-Technologies, Tokyo, Japan) (JSM-6701F; JEOL, Tokyo, Japan). The specimens were observed at accelerating voltages of 5 and 15 kV after the Au sputter coating.

The three-dimensional morphology of ZrO_2_/Ti surface was observed using AFM (Nanosurf Easyscan 2, Nanosurf AG; Liestal, Switzerland). The tapping mode was employed using a TapAL-G cantilever (Budget sensors, Bulgaria; resonance frequency = 190 kHz, spring constant = 48 N/m). The average arithmetic roughness of the three-dimensional surface (Sa) was calculated within the 25 μm × 25 μm area of the AFM images. The sandblasted rough Ti surface was observed using SEM and AFM in the same manner. Five specimens were tested under each condition.

The wettability of each sample was determined based on its contact angle with distilled water. The volume of the water droplet was measured using a contact angle meter (DMe-201, Kyowa Interface Science Co., Ltd., Tokyo, Japan) with a 0.5 µL drop for the disk-type sample and a 0.2 µL drop for the cylinder-type sample. For both disk and cylinder types, the water droplets were placed on the center of each specimen. 1 s measurements on each surface were taken under 25 ± 1 °C and 46% humidity. Ten specimens for each condition were measured.

EDX (Miniscope TM3030, HITACHI/Xflash MIN SVE; Bruker, Kanagawa, Japan) at an accelerating voltage of 15 kV was used to confirm the presence of Ti or Zr atoms on the Ti and ZrO_2_/Ti cylinders. The cross-sectional images were analyzed. Cross-sectional samples were obtained by embedding the specimen in epoxy resin, allowing it to cure, and then cutting the specimen vertically through the center using a cutting machine, namely, the EXAKT Cutting-Grinding system (EXAKT Cutting-Grinding System and BS-300CP band system EXAKT Apparatebau, Norderstedt, Germany).

The atomic elements present on the surface of SLA/Ti and ZrO_2_/Ti were analyzed using XPS (JPS-9030; JEOL, Tokyo, Japan), which X-ray source is Mg Kα and power is 12 kV × 5.0 mA. The measured binding energies were corrected using the C 1s orbital energy value of 284.5 eV.

Crystal structure of the zirconia thin film deposited on Ti disk was characterized using XRD measurement with an attachment incidence angle θ = 0.3° (SmartLab; Rigaku, Tokyo, Japan). Cu Kα is the X-ray source (45 kV × 200 mA).

### 2.3. Animal Implantation Experiment

This animal study was approved by the Animal Experimental Ethical Guidelines of the Tsurumi University School of Dental Medicine (certificate numbers 23A047 and 24A033).

**Figure 3:**
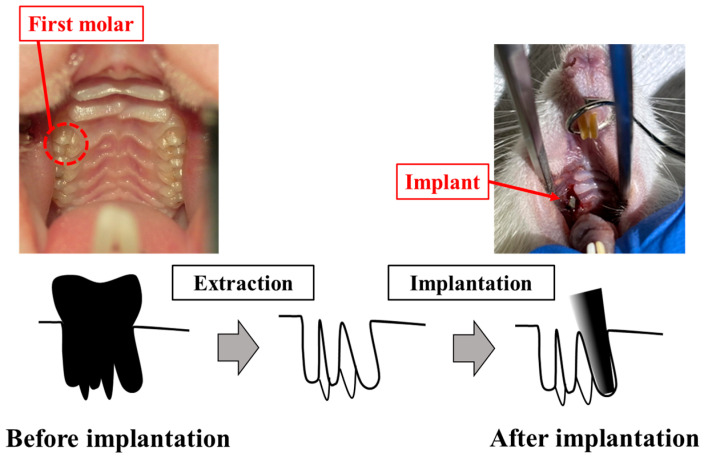
Process for animal experiment.

The process for animal experiment is shown in Figure 3. Implants were inserted in the tooth socket of the extracted maxillary teeth of male Wistar rats (6-week-old, 180–200 g). The rats were housed at the animal experimental facility of Tsurumi University at 5 weeks of age in order to allow them to become accustomed to the experimental environment. During the experiments, two rats were housed per cage. The temperature was controlled at 20–25 °C, with a 12-h light/dark cycle and free access to water and powdered food. Eight rats were housed in four cages. The rats were randomly divided into two groups: ZrO_2_/Ti and SLA/Ti. Each rat received one implant, for a total of 8 implants in 8 rats. Four ZrO_2_/Ti and four SLA/Ti implants were placed for 3-week implantation periods (*n* = 4, sample size was determined by the instruction of the Animal Experimental of the Tsurumi University School of Dental Medicine). At the same time, the opposing tooth was extracted. There were no animal exclusions.

The implants were sterilized with ethylene oxide gas before implantation. The implants were inserted into the extraction sockets of the maxillary first molars of rats as previously reported [12,18,19]. Surgery was performed under general anesthesia, with ketamine hydrochloride administered intraperitoneally. (0.6 mg/kg; Daiichi Sankyo Propharma Co. Ltd., Tokyo, Japan). Sedation and analgesia were induced by injecting medetomidine hydrochloride (0.3 mg/kg; Nippon Zenyaku Kogyo Co. Ltd., Fukushima, Japan). Rats were awakened using atipamezole hydrochloride (0.15 mg/kg; Nippon Zenyaku Kogyo Co., Ltd., Fukushima, Japan). The right maxillary first molar was carefully extracted using osteoclastic forceps. Four ZrO_2_/Ti and four SLA/Ti implants were press-fit-inserted using root canal tweezers. The contralateral tooth was extracted in order to avoid occlusal pressure load on the implant body. After surgery, antibiotics (latamoxef sodium, 0.01 mg/kg; Shiomalin, SHIONOGI Co., Ltd., Osaka, Japan) were subcutaneously injected.

### 2.4. Histological Observation and Histomorphometric Analysis

The implants were not protected with caps. Three weeks after implantation, the implants, including the surrounding bone and soft tissues, were excised to observe the early response of the surrounding tissue according to previous reports [8,13,20]. Each specimen was first stored in a 10% neutral-buffered formalin solution for fixation and then dehydrated by sequential immersion in 70%, 80%, 90%, 96%, and 100% ethanol. Subsequently, each specimen was embedded in methyl methacrylate resin. After resin polymerization, a non-decalcified thin section was prepared by a cutting and polishing procedure using the EXAKT cutting–grinding system [20]. The cut sections were placed parallel to the implant axis. Each section was polished with waterproof paper (800, #1200, #2000, #2400, and #4000) and the thickness was finally approximately 70 to 80 μm. One implant was divided at the midline and two sections were obtained.

**Figure 4:**
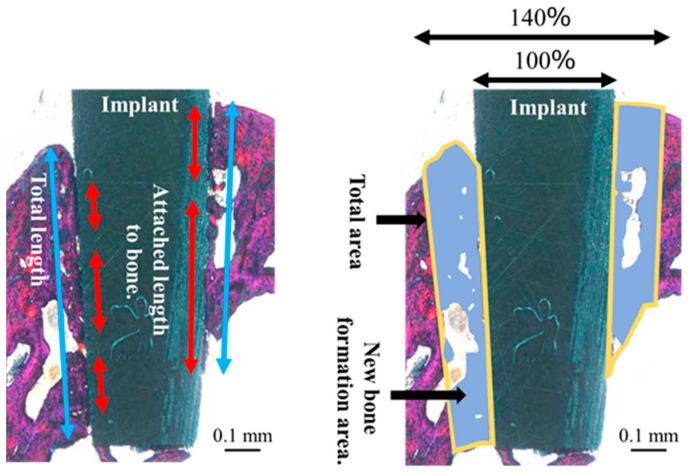
Schematic diagram of the bone to implant contact and bone mass calculation measured within regions of interest.

Histological evaluation of each section was performed randomly. After double staining with methylene blue and basic fuchsin, the sections were observed under a light microscope (Eclipse Ni; Nikon Corp., Tokyo, Japan; ×40 and ×100 magnification). The bone response toward the ZrO_2_/Ti and SLA/Ti implants was evaluated histologically and histomorphometrically. The bone-to-implant contact (BIC) ratio and bone mass (BM) were calculated using histomorphometric analysis. BIC was defined as the percentage of the length of the implant surface in direct contact with the bone, and BM was calculated as the percentage of newly formed bone around the implant. The BM and BIC were measured within the regions of interest (ROI), as determined in accordance with a previous study [21], as illustrated in Figure 4. The ROI is a box on a section whose vertical length is the length of the implant body and whose width is the width of the upper end of the implant plus 20% on both sides. The vertical and horizontal lengths of each section differed depending on the implant insertion direction and the cutting position of the sample; therefore, they were determined by measuring each sample individually. The BIC ratio and BM values were calculated using the results obtained from the image analysis (WinROOF, Visual System Division Mitani Co., Ltd., Tokyo, Japan).

**Figure 5:**
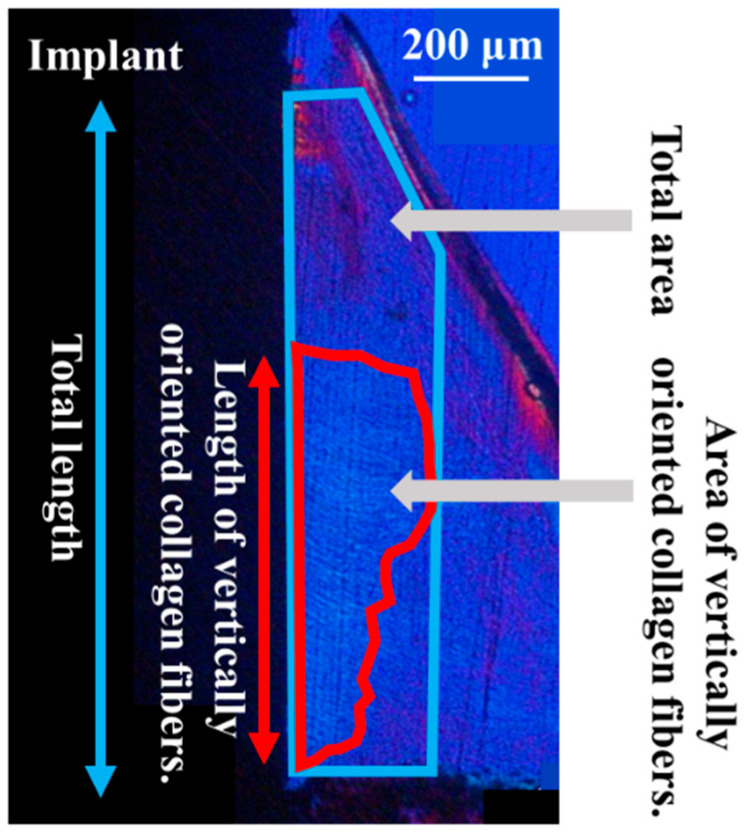
Schematic diagram of the total length or area of soft tissue attachment to the implant surface by regions of interest.

The orientation of the peri-implant soft tissue relative to the collagen fiber bundles was observed under a polarized light microscope (ECLIPSE LV100N POL, Nikon, Tokyo, Japan) to determine the soft tissue response toward the ZrO_2_/Ti and SLA/Ti implants. Histomorphometric evaluations were performed using the WinROOF image analysis system, based on a previous study [8]. The ROI was defined as shown in Figure 4. Measurements were taken only in the soft tissue area. Figure 5 shows a schematic drawing of the measurement of the total length or area of the vertically oriented collagen fibers around the implant. The total length or area of the length or area of soft tissue attachment toward the ZrO_2_/Ti and SLA/Ti implant surfaces was calculated. The length or area of the attachment site where the collagen fibers were oriented perpendicularly toward ZrO_2_/Ti and SLA/Ti was calculated. Vertical attachment ratio was calculated as the ratio of the vertical length or area to the total length or area. This value is denoted as the length or area as a measure of soft tissue integration.

### 2.5. Statistical Measurement

The sample size for each experiment was determined according to our previous reports [8]. Statistical significances were determined using SPSS version 25 (IBM Japan, Ltd., Tokyo, Japan). First, the normality of the data distribution was evaluated using the Shapiro–Wilk test, and the homogeneity of variances was assessed using Levene’s test. Data satisfying both normality and homogeneity of variances were analyzed using an unpaired *t*-test. Conversely, when the data did not satisfy normality and/or homogeneity of variances, the Mann–Whitney U test was employed. Statistical significance was determined as *p* < 0.05, and numerical data were expressed as means and standard deviations, and values were expressed as means (standard deviation).

## 3. Results

### 3.1. Surface Characterization

**Figure 6:**
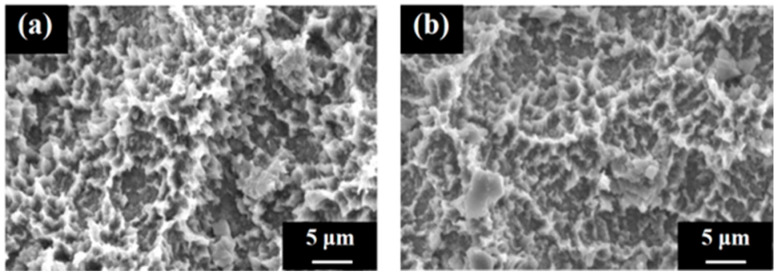
Scanning electron microscopy images of the cylinder samples. Image of the (**a**) SLA/Ti sample and (**b**) ZrO_2_/Ti sample.

Figure 6 shows the surface appearances of the SLA/Ti and ZrO_2_/Ti cylinders obtained using SEM. No difference was observed in the surface appearance of the SLA/Ti and ZrO_2_/Ti cylinders. Both surfaces exhibited microscale dimples formed by irregular roughness on the rough sandblasted surfaces. Furthermore, no peeling or defects of the ZrO_2_ coating was observed.

**Figure 7:**
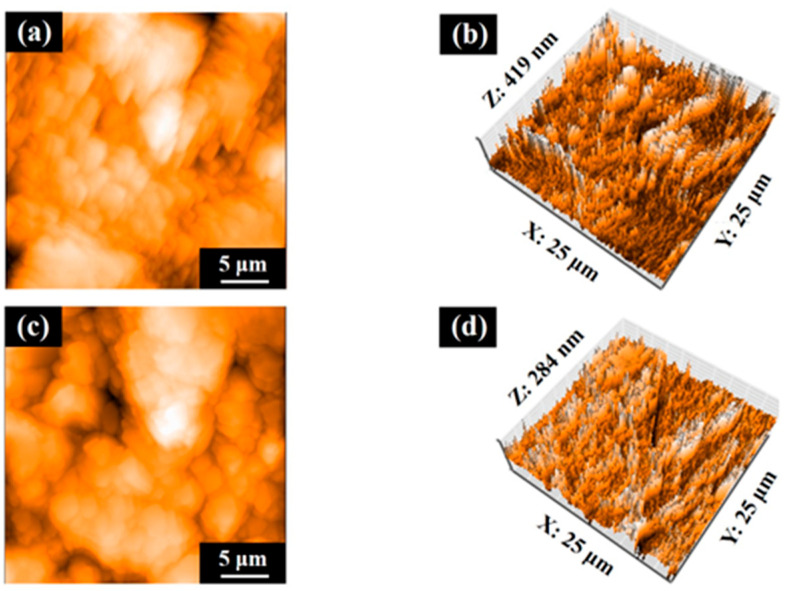
Atomic force microscopy profiles of cylinder samples. Images of (**a**,**b**) SLA/Ti and (**c**,**d**) ZrO_2_/Ti.

**Figure 8:**
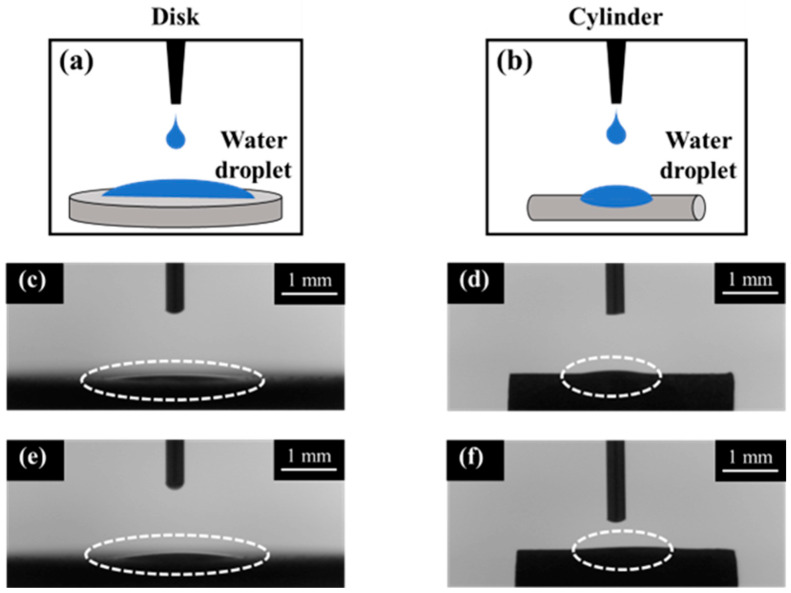
Contact angle measurement images of disk and cylinder samples. Illustration of contact angle measurements were also shown. (**a**) disk and (**b**) cylinder. Dotted circles show the water droplet on each sample. SLA/Ti (**c**) disk and (**d**) cylinder. ZrO_2_/Ti (**e**) disk and (**f**) cylinder. The dash lines indicate the water droplet.

Figure 7 shows the AFM profiles of the SLA/Ti and ZrO_2_/Ti cylindrical surfaces. The contact angle measurements for the disk and cylinder on SLA/Ti and ZrO_2_/Ti samples are shown below (Figure 8). For the arithmetic average roughness (Sa), the data satisfied normality but not homogeneity of variances. Therefore, the Mann–Whitney U test was employed, and no significant differences were showed in Sa among the SLA/Ti and ZrO_2_/Ti cylinders (lower confidential limit: −277.99552, upper confidential limit: 240.95952, *p* = 0.841). For the contact angles of the disk specimens, the data satisfied both the normality and homogeneity of variance; therefore, an unpaired *t*-test was performed. Significant difference existed between SLA/Ti and ZrO_2_/Ti (lower confidential limit: −4.13868, upper confidential limit: −2.22132, *p* < 0.001). For the contact angle of the cylinder specimens, the data showed neither normality nor homogeneity of variances. Thus, the Mann–Whitney U test was applied. No significant differences were existed between the SLA/Ti and ZrO_2_/Ti cylinders (lower confidential limit: −4.95462, upper confidential limit: 6.53462, *p* = 0.684). (Table 1).

**Figure 9:**
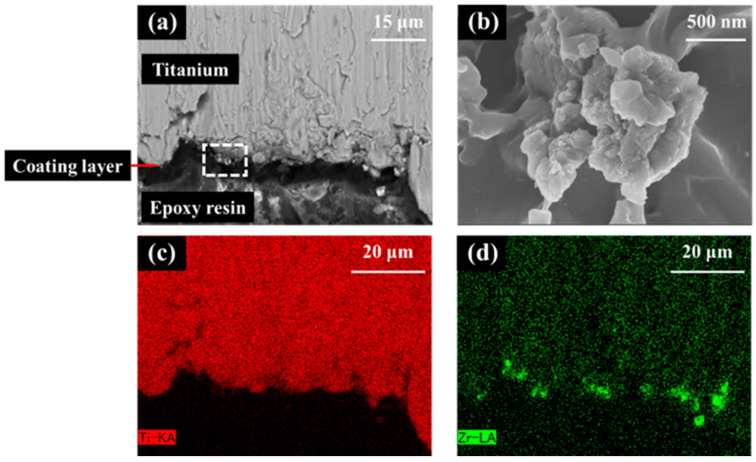
Scanning electron microscopy (SEM) image and X-ray spectroscopy (EDX) analysis of the zirconia-coated cylinder surface. (**a**) Low and (**b**) high images of the ZrO_2_/Ti sample SEM images. (**b**) is an enlarged photograph of the area enclosed in the box in (**a**). (**c**) Ti and (**d**) Zr mapping of EDX.

In the SEM images of the interface between the titanium substrate and zirconia coating layer for the zirconia-coated cylinder surface (Figure 9), the coating layer can be discerned and the crystal structure of zirconia can be confirmed. EDX analysis confirmed the presence of Zr on the Ti surface.

**Figure 10:**
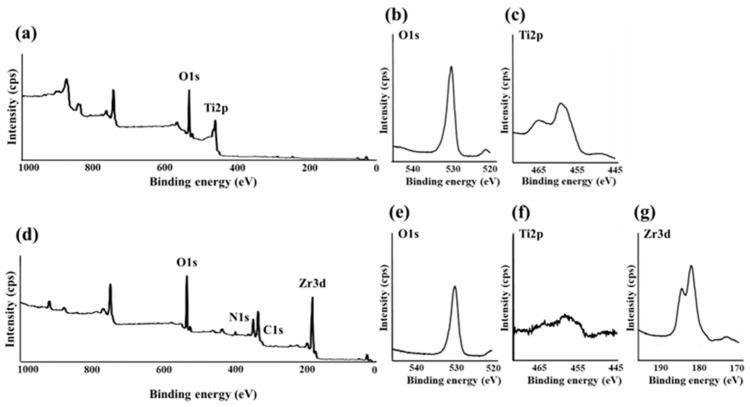
(**a**) Broad spectrum of the SLA/Ti surface measured using X-ray photoelectron spectroscopy. (**b**) O1s and (**c**) Ti2p spectra of SLA/Ti surfaces. (**d**) Broad spectrum of the ZrO_2_/Ti surface measured using X-ray photoelectron spectroscopy. (**e**) O1s, (**f**) Ti2p, and (**g**) Zr3d spectra of ZrO_2_/Ti surfaces.

In the XPS analysis of the Ti disks, SLA/Ti showed O1s and Ti2p peak from TiO_2_, and no Zr peak (Figure 10a–c). ZrO_2_/Ti specimen also showed the O1s peak at 530.2 eV owing to TiO_2_ and ZrO_2_ (Figure 10d,e). But Ti2pwas hardly detectable (Figure 10f). Peaks attributed to Zr3d were observed at 182.2 and 184.5 eV (Figure 10g). This confirms the presence of a zirconia coating on the Ti implant surface.

**Figure 11:**
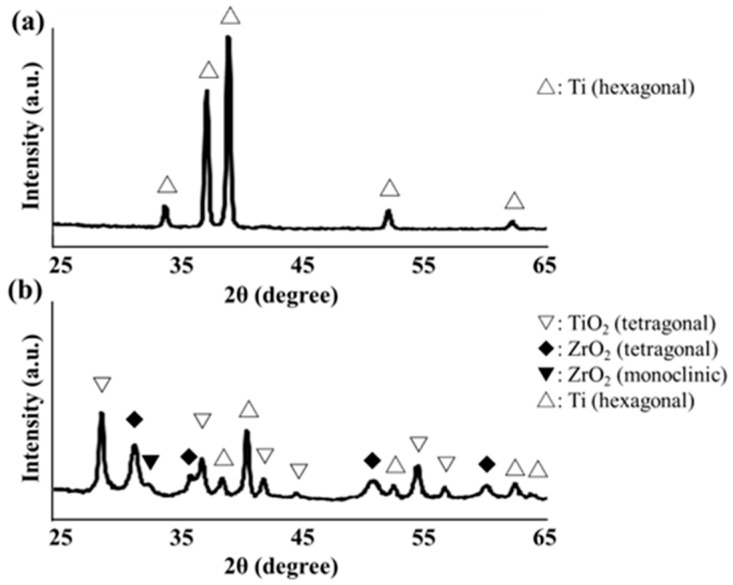
(**a**) X-ray diffraction profile of SLA/Ti disk surface. (**b**) X-ray diffraction profile of ZrO_2_/Ti disk surface.

Figure 11 shows the XRD profiles of the SLA/Ti surface and zirconia films on the SLA/Ti disks. For SLA/Ti disk, only hexagonal Ti peak was identified (Figure 11a). A deposited coating with zirconia structures was confirmed for ZrO_2_/Ti specimen. Corresponding to monoclinic zirconia, a peak was observed at 31.5°, whereas peaks at 30.3°, 35.3°, 50.4°, and 60.2° were observed for tetragonal zirconia. Ti peaks were also observed.

### 3.2. Histological and Histomorphometric Evaluations

The rats remained healthy throughout the experimental and healing periods. No clinical signs of unfavorable tissue reactions, such as inflammation, were observed when the animals were euthanized, and all implants remained in situ.

**Figure 12:**
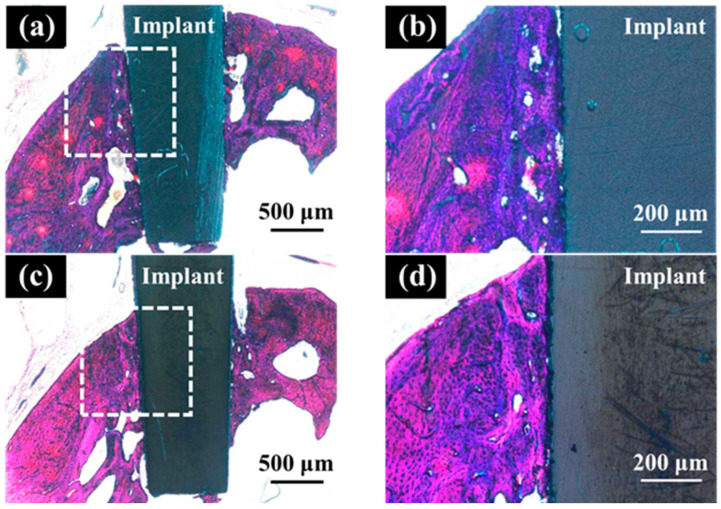
Histological appearance of the hard tissue 3 weeks after implantation into a rat tooth extraction socket. SLA/Ti (**a**) low magnification and (**b**) high magnification. ZrO_2_/Ti (**c**) low magnification and (**b**) high magnification.

In the optical microscopic image of hard tissue around SLA/Ti and ZrO_2_/Ti implants 3 weeks after placement, osseointegration of the titanium implants with bone was confirmed in all specimens, with no epithelial cell downgrowth observed (Figure 12). New bone was formed surrounding the implants three weeks after implant placement. Bone remodeling and mature bone formation were observed. Overall, more new bone was observed with ZrO_2_/Ti implants than with SLA/Ti implants.

Table 2 lists the results of the BIC ratio and BM values 3 weeks after implantation. There was no significant difference in the BIC ratio between ZrO_2_/Ti implants and SLA/Ti implants (lower confidential limit: 3.66809, upper confidential limit: 29.79309, *p* = 0.105). However, the BM values of ZrO_2_/Ti implants were significantly higher than those of SLA/Ti implants (lower confidential limit: 6.022217, upper confidential limit: 28.70708, *p* = 0.010). The normality of the data distribution and the homogeneity of variances for BIC and BM measurements were confirmed. Thus, the data were analyzed using an unpaired *t*-test.

**Figure 13:**
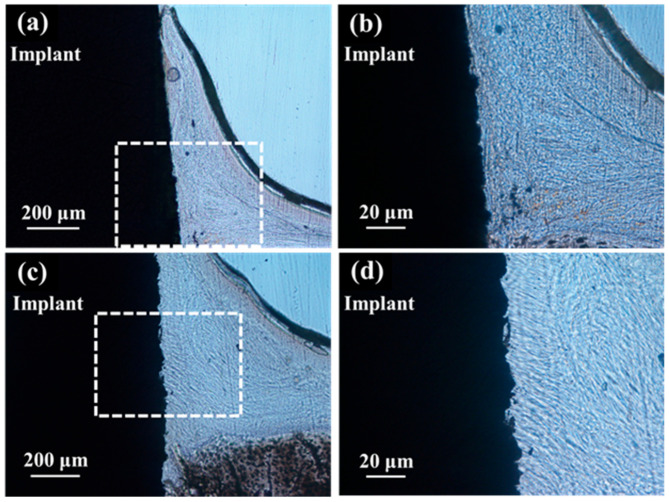
Histological appearance of the soft tissue 3 weeks after implantation into a rat tooth extraction socket. SLA/Ti (**a**) low magnification and (**b**) high magnification. ZrO_2_/Ti (**c**) low magnification and (**d**) high magnification. In the optical microscopic images, there are no gaps between the soft tissue and the Ti implant for both SLA/Ti and ZrO_2_/Ti implants. Even at higher magnification, the attachment of soft tissue is without gap formation.

In the optical microscopic images of the palatal soft tissue SLA/Ti and ZrO_2_/Ti implants 3 weeks after placement, no gaps were noted between the Ti implant and the soft tissue in any specimen (Figure 13).

**Figure 14:**
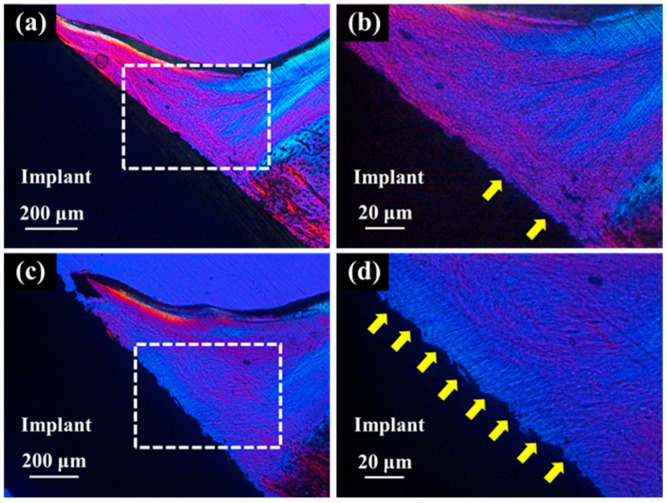
Polarized light micrographs of soft tissues around SLA/Ti and ZrO_2_/Ti. (**a**,**b**): SLA/Ti implant. (**c**,**d**): ZrO_2_/Ti implant. (**b**,**d**) are higher magnification images of (**a**,**c**), respectively. Yellow arrow: vertically oriented collagen fibers. Collagen fibers exhibit birefringence due to their anisotropic structure. In a polarizing microscope, the color of the transmitted light indicates the direction or orientation of the collagen fibers, allowing visual observation of their orientation. Thus, collagen fibers parallel to Ti implant and those to perpendicular showed different colors.

Polarized light microscopy of the palatal soft tissue for the ZrO_2_/Ti and SLA/Ti implants revealed that the ZrO_2_/Ti implants had more collagen fibers oriented perpendicular to the surface of the implant than SLA/Ti implants. (Figure 14). Conversely, SLA/Ti exhibited collagen fibers that were short and perpendicular to the Ti implant surface; however, more collagen fibers ran parallel to the implant body around the Ti implant.

Table 3 lists the results of the histomorphometric analyses of the vertically oriented collagen fibers at 3 weeks after implantation. The length of the surrounding perpendicularly oriented collagen fibers relative to the implant body was significantly longer in the ZrO_2_/Ti implants than in the SLA/Ti implants (lower confidence limit: 31.65618, upper confidence limit: 65.37882, *p* < 0.001). The area of the vertically oriented collagen fibers around the ZrO_2_/Ti implants was significantly larger than that around the SLA/Ti implants (lower confidence limit: 19.58019, upper confidence limit: 52.96981, *p* = 0.002). Normality of the data distribution and homogeneity of variances for histomorphometric measurements of vertically oriented collagen fibers were confirmed. Therefore, data were analyzed using unpaired *t*-tests.

## 4. Discussion

The null hypothesis was rejected. The ZrO_2_-coated Ti implants showed significantly higher BM values and greater lengths and areas of collagen fibers oriented perpendicularly to the implant body than Ti implants. Several studies have compared the Ti and Y-TZP implants. Y-TZP implants have been reported to have better soft-tissue compatibility and suppress bacterial adhesion than Ti implants [22]. Conversely, Ti implants have been reported to result in better healing of the surrounding soft tissue than Y-TZP implants [23]. Furthermore, insufficient data exists from animal experiments to substantiate these findings [24]. Thus, reports on Y-TZP implants are inconsistent and many unknowns remain.

In this study, we have deposited a thin ZrO_2_ coating on Ti implants while maintaining their surface structures. The animal model used in this study is an original and highly useful model that can reproduce biologically and physically harsh oral environment [12,25]. This is the first study to simultaneously examine the effects of ZrO_2_-coated implants on hard and soft tissues in an oral environment.

Over the past few years, attention has focused on the surface treatment of Y-TZP implants. However, it remains unclear which type of surface treatment and surface morphology is superior [26]. The molecular precursor method used in this study is a wet method that uses metal complexes to deposit various functional metal oxides into ultra-thin films with a thickness of less than 1 μm [27]. This simple method involves applying a solution to a substrate, followed by heat treatment. Because ethanol was used, it was possible to form a uniform thin film with excellent substrate conformability [16,17,28]. Tokunaga et al. [13] reported that the molecular precursor method can form thin films while maintaining the rough surface of disk-shaped substrates. However, reports on the coatings of dental implants with complex surface shapes are lacking. The SEM and AFM results showed no morphological differences when compared to the control, indicating that coating with molecular precursors is possible without affecting the surface shape of substrates with complex shapes, such as dental implants.

Zirconia crystals were observed on the ZrO_2_/Ti surface in the SEM images. The uniform presence of zirconia in the EDX analysis was attributed to the peeling of the coating during sample preparation. Considering that the AFM results showed no difference in the surface roughness, Sa value, between the SLA/Ti and ZrO_2_/Ti, coating with the molecular precursor method did not affect the surface shape. The XRD results confirm the formation of a tetragonal zirconia thin film. Generally, in bulk materials, as the temperature decreases, the crystal structure changes from cubic to tetragonal and monoclinic [29,30]. In this study, the thin film was influenced by the substrate, resulting in lower crystallization and phase transition temperatures than those of bulk zirconia, and tetragonal crystals were identified. However, the present analysis did not provide any quantitative data on the crystal structure. Therefore, a more detailed quantitative analysis of the zirconia crystal structure should be conducted.

SLA renders the treated material hydrophilic [31]. In this study, the contact angles of the cylindrical samples were greater than those of the disk-shaped samples. This is because the surface tension of water was greater on the cylinder than on the disk-shaped sample owing to the surface shape. Considering that there was no significant difference between the coated and non-coated samples of the same type, that is, disk or cylinder, it was possible to produce samples with hydrophilicity equivalent to that of the Ti implants currently used in clinical practice by applying zirconia coating using the molecular precursor method.

No significant difference in the BIC was observed between ZrO_2_/Ti and SLA/Ti. In contrast, ZrO_2_/Ti exhibits a significantly higher BM than SLA/Ti. The reason why BM is significantly different even though the contact between the bone and implant within the ROI, namely BIC, remains significantly unchanged, is that the zirconia-coated implant acts on the surrounding bone tissue, including osteoblasts, resulting in more active bone formation near the implant interface, which is not in contact with the implant. The details are unclear; however, if, for example, the evaluation was performed at different implantation periods, this may have been confirmed. Tokunaga et al. [13] embedded rectangular plate-shaped specimens into the femurs of rats and evaluated them after two and four weeks. They reported significant differences in BIC and BM two weeks after implantation, but no differences after four weeks. Therefore, future studies should therefore investigate BIC and BM over various implantation periods to better understand early healing processes.

Generally, a rough titanium surface is not suitable for soft tissue interactions, whereas a smooth surface is beneficial for soft tissue attachment [32]. Directionally microgrooved surfaces have been reported in the past to enhance soft tissue attachment by regulating the fibroblast orientation [33,34]. In contrast, the present study revealed that thin-film zirconia surfaces deposited on sandblasted titanium can control the alignment of collagen fibers. A sandblasted surface is a randomly roughened surface without regular direction. In the present soft tissue evaluation, quantitative evaluation using image analysis revealed that in ZrO_2_/Ti, numerous collagen fiber bundles were found perpendicular to the implant body, similar to natural teeth. Conversely, in the SLA/Ti implants, numerous collagen fiber bundles were found parallel to the implant.

The molecular precursor method used in this experiment maintained the surface shape of the base material, revealing no morphological differences between the ZrO_2_/Ti and SLA/Ti samples. Therefore, the chemical properties of zirconia may influence the differences in the orientation of the collagen fiber bundles. Barker et al. [35] reported that SLA treatment was beneficial for soft tissue attachment using 3D oral mucosal models. Yan et al. [36] in their study reported that the preparation of a microgroove coating on zirconia mediates the glycolysis of fibroblasts and improves the sealing of connective tissue. Hsu et al. [37] reported that a rough zirconia surface strengthened the fixation of type I collagen fibers in a cell assay. In these cases, the surface shape of zirconia has been reported to be favorable for soft tissue attachment. The results of this study suggest that the chemical properties of zirconia, rather than its morphology, directly affect the arrangement of collagen fiber bundles. The perpendicular orientation of collagen fibers produces a tight seal on the implant by collagen fibers and prevents peri-implantitis. Lee et al. reported healing patterns of the mucosal seal on zirconia implants compared with titanium implants [2]. Cell proliferation assays and implantation into the maxillary first molar area of the mice were performed. The proliferation rate of the human periodontal ligament fibroblasts on the zirconia surface was higher than that on Ti surfaces. Zirconia implants showed less elongation of the peri-implant epithelium than Ti. The expression of laminin-332 participates in the elongation of the peri-implant epithelium. The ratio between the connective tissue and epithelium in zirconia was found to be almost the same as that in natural teeth. Ti had a significantly lower ratio of connective tissue. A systematic review of the fibroblast response to zirconia and titanium implant abutments reported that zirconia exhibited comparable or superior efficacy in promoting the proliferation of human gingival fibroblasts compared with titanium [38]. The detailed mechanism by which the present zirconia surface induces the vertical orientation of collagen fibers is not clear; the above-mentioned factors, such as laminin-332 expression, influence the orientation of collagen fibers. In the future, we will investigate the differences in the expression of genes, proteins, and signals in the surrounding soft tissues between ZrO_2_/Ti and SLA/Ti. The difference in implant shape is another factor that influences the surrounding tissue response. In dental clinics, most implants are of the screw type. In this study, we used a cylindrical implant to evaluate only the influence of surface chemistry on tissue response. Evaluation of zirconia coatings on screw-type implants will be further investigated. Surface roughness is another factor that influences tissue response. Sa value for commercially available SLA titanium implants is reported to be approximately 1.5 μm [39]. In contrast, the Sa values of the present specimens were less than 1.5 μm. This was due to the difference in the particle size of the sandblasted powder. The diameter of the sandblasting powder for commercially used SLA implants is 180–250 μm, while the diameter of the sandblasting powder used in this experiment was 180 μm. A rougher surface produces a better bone response. However, although some cell experiments have confirmed that rough surfaces are beneficial for the soft tissue response, as described above [35,37], the influence of surface roughness on soft tissues, particularly collagen fiber alignment, after implantation in vivo has not been elucidated. This will be investigated in future studies.

Lee et al. histologically analyzed implantations at eight weeks [2]. It was difficult to predict long-term soft tissue sealing based on our 3 weeks’ observation. The limited sample size and absence of functional loading may have limited the results of this study. Longer implantation periods in the loaded condition and a larger sample size are therefore necessary to achieve clinical efficacy.

## 5. Conclusions

Within the limitations of this brief animal experiment, zirconia-coated titanium implants showed bone formation and collagen fiber orientation patterns, thus suggesting a favorable tissue response when compared to uncoated titanium. Future experiments are needed to evaluate the histology of zirconia-coated screw-type implants with a longer duration under load and a larger sample size.

## Figures and Tables

**Table 1:** Three-dimensional surface roughness (Sa) and contact angle of SLA/Ti and ZrO_2_/Ti.

	Sa (nm)	Contact Angle (°)	
	Cylinder	Disk	Cylinder
SLA/Ti	716.2 (212.4) ^a^	7.3 (0.9) ^b^	24.9 (2.0) ^d^
ZrO_2_/Ti	679.7 (66.4) ^a^	4.1 (1.1) ^c^	25.7 (7.9) ^d^

Values in brackets are standard deviation. The same letters indicate no significant differences (*p* > 0.05).

**Table 2:** Percentage of calculated BIC ratio and BM values.

Specimen	BIC Ratio (%)	BM (%)
SLA/Ti	68.6 (11.2) ^a^	59.7 (5.8) ^b^
ZrO_2_/Ti	81.7 (7.9) ^a^	77.0 (7.2) ^c^

Values in brackets are standard deviation. The same letters indicate no significant differences (*p* > 0.05).

**Table 3:** Histomorphometrical measurements of vertically oriented collagen fibers.

Specimen	Length (%)	Area (%)
SLA/Ti	18.8 (10.0) ^a^	16.8 (2.6) ^c^
ZrO_2_/Ti	67.3 (9.5) ^b^	53.1 (13.4) ^d^

Values in brackets are standard deviation. Different letters indicate significant differences (*p* < 0.05).

## Data Availability

The original contributions presented in the study are included in the article, and further inquiries can be directed to the corresponding author.

## Abbreviations

The following abbreviations are used in this manuscript:

Y-TZP	partially stabilized zirconia
SLA	sandblasted and acid-etched
SEM	scanning electron microscopy
AFM	atomic force microscopy
EDX	X-ray spectroscopy
XPS	X-ray photoelectron spectroscopy
XRD	X-ray diffraction
BIC	Bone-to implant contact
BM	bone mass
ROI	regions of interest
